# Addressing the caste system in U.S. healthcare in the era of COVID-19

**DOI:** 10.1186/s12939-020-01298-x

**Published:** 2020-10-19

**Authors:** Karthik Sivashanker, Cheri Couillard, Jennifer Goldsmith, Normella Walker, Sunil Eappen

**Affiliations:** 1 Department of Quality and Safety, Brigham and Women’s Hospital, Boston, USA; 2 Department of Diversity, Inclusion, and Experience, Brigham and Women’s Hospital, Boston, USA; 3 Trauma Therapist, Brandon Residential Treatment Center, Natick, USA; 4 Division of Global Health Equity, Brigham and Women’s Hospital, Boston, USA; 5 Department of Medicine, Brigham and Women’s Hospital, Boston, USA; 6 Brigham and Women’s Hospital, Boston, USA

**Keywords:** Health, Equity, Racism, Caste

## Abstract

In healthcare, we find an industry that typifies the unique blend of racism, classism, and other forms of structural discrimination that comprise the U.S. caste system—the artificially-constructed and legally-reinforced social hierarchy for assigning worth and determining opportunity for individuals based on race, class, and other factors. Despite myths of meritocracy, healthcare is actually a casteocracy; and conversations about racism in healthcare largely occupy an echo chamber among the privileged upper caste of hospital professionals. To address racism in healthcare, we must consider the history that brought us here and understand how we effectively perpetuate an employee caste system within our own walls.

## Main text

The COVID-19 pandemic and resurgence of Black Lives Matter amidst continued police killings of unarmed Black men and women, have led us to a historic moment. We are collectively grappling with the recognition of structural racism that pervades our society, and with the significance of systems that normalize and elevate whiteness while diminishing black and brown experience.

This awakening is also happening in hospitals across the country. In healthcare, we find an industry that typifies the unique blend of racism, classism, and other forms of structural discrimination that comprise the U.S. caste system—a framework from Pulitzer-prize winning author Isabel Wilkerson that describes the artificially-constructed and legally-reinforced social hierarchy for assigning worth and determining opportunity for individuals based on race, class, and other factors. To address racism in healthcare, we must consider the history that brought us here and understand how we effectively perpetuate an employee caste system within our own walls.

### The creation of caste in the U.S

In codifying U.S. slavery in the 1700’s, a privileged elite created racist policies and practices to preserve a system of wealth and social inequality, establishing concepts of ‘white’ and ‘black’ formally in US law. Rich and poor whites alike were conferred a special status, while blacks were relegated to slavery. Thus, the elite class reduced the risk of poor whites from uniting and rebelling in common cause with Black enslaved peoples. From Reconstruction forward, racism has defined and influenced almost all of our social and economic policies in one way or another—from housing, education and employment discrimination, to mass incarceration, medical experimentation, lynchings, mob violence, and state-sanctioned police brutality. Thus, through systematic oppression and deprivation, Black people have been relegated to the lowest socioeconomic rank of the U.S. caste system. We see other groups similarly treated. The genocide, forced assimilation and relocation of indigenous and Native American people to reservation lands, and the ongoing xenophobic treatment of undocumented brown people and immigrants, are other examples.

### The caste system in U.S. healthcare

Healthcare exemplifies this manufactured hierarchy whereby white (and cisgender, non-disabled, affluent, male) employees are assigned the highest value and preferential treatment over others. As a result, employees of color remain grossly overrepresented in low-wage essential jobs such as cleaning patient rooms, transporting patients, and preparing food, while being overseen by mostly white supervisors; and underrepresented among hospital boards, executive leaders, and managers, with little change over the last decade [1]. This labor hierarchy descends from eras when low-skilled jobs were the only ones available to people of color and echoes a lack of generational wealth. Roughly 75% of medical school graduates come from the two highest household income quintiles and Black students entering medical school must contend with much higher levels of educational debt (62%) as compared to white students (36%) [2]. In additional to economic inequities, these same clinicians routinely face racist micro- and macro-aggressions from staff and patients.

Despite myths of meritocracy, healthcare is actually a casteocracy; and conversations about racism in healthcare largely occupy an echo chamber among the privileged upper caste of hospital professionals. Academic activities (research, publications, Grand Rounds) and hospital initiatives like social justice committees, typically cater to a salaried, educated, primarily English-speaking workforce. Rarely are hourly employees who are at the bottom of the socioeconomic hierarchy meaningfully included in this work.

### COVID-19 and employee fracturing

Under fear and anxiety associated with COVID-19, fractures emerged among employee groups at healthcare institutions. Nurses filed safety reports, questioning why doctors waited outside patient rooms while nurses themselves provided hands-on care. Clinicians questioned the thoroughness of room cleaning and timeliness of transportation, while environmental services and transportation workers reported resistance from some clinical staff in sharing scarce personal protective equipment (PPE). Staff redeployment led to questions about why some had to report in-person and others were allowed to work virtually. Themes emerged from safety and experience reports around fragmentation, power displays, top-down decision-making, information hoarding, and breakdowns in communication. These problems are pervasive across many hospital systems and reflect longstanding mistrust and tension between labor groups at different socioeconomic levels.

### Challenges for low wage healthcare workers

Low wage healthcare workers have long faced poverty even despite their employment. As of 2017, people of color were concentrated in the lowest wage healthcare positions. Among them, 34% of non-white healthcare workers earned less than $15 per hour and earned less on average than their white colleagues [3, 4]. Among female healthcare workers and their children, 1.7 million lived below the poverty line and many lacked healthcare coverage and/or relied on public assistance to survive [3, 4]. In the context of the COVID-19 crisis, many of these low-income workers struggled to find or afford childcare, many saw job losses in their families putting them at risk while still employed and some were furloughed or experienced reduced hours at hospitals across the country. These same employee groups also contracted COVID-19 at higher rates compared to those in higher income brackets, reflecting challenges with socially distancing often due to crowded housing conditions, much higher use of public transportation, multiple worker households, and essential frontline jobs.

### Brigham Health and COVID-19: a case example

At Brigham Health, these inequities were identified early in the pandemic through a robust incident command infrastructure that systematically incorporated equity-based reporting of both patient and employee data [5]. We discovered that some of our lower paid employee groups like environmental and food services were testing positive for COVID-19 at up to 10 times the rates of higher wage frontline workers such as physicians and nurses. Based on infectious disease tracers and a comparison of employee and community-level data, we saw that COVID-infection rates between employee groups reflected transmission patterns in the community. Employees from lower-income neighborhoods and neighborhoods of color tested positive at higher rates often mirroring the red-lined maps from generations ago.

We recognized that these employees were unlikely to read the barrage of email COVID-19 updates since their workdays are not spent in front of computers. We observed that email messages were exclusively offered in English and often written in a technical rather than practical style. Thus, even with the need to de-densify and limit group meetings, hospital leadership felt it was essential to offer clear accessible information face-to-face with frontline staff.

### Bridging the caste divide

From April – June 2020, we hosted socially-distanced small group sessions for workgroups including environmental services, security, materials management, food services, transport, and patient care and medical assistants. Substantively, the sessions acknowledged frontline workers’ contributions and expressed appreciation, transparently shared information on COVID-19 infection rates by groups and geography, reinforced best practices to avoid COVID-19 transmission, presented human resource policies, and made accessible resources such as housing, finance, and domestic violence assistance. We encouraged COVID-19 testing for symptoms, and provided reassurance around job, income, and personal leave time if employees tested positive for COVID-19. Childcare costs were significantly subsidized by the hospital for lower income staff and made free for internal centers, and equitable access to PPE and COVID-19 testing was ensured.

Sessions were held around shift change including meetings at 6:30 am and 11:30 pm, times when the lowest wage workers (not salaried employees) typically fill the halls of the hospital. One hundred two in-person sessions were attended by 1075 employees. Over 55 staff across multiple disciplines served as facilitators. Documents were translated into five different languages and sessions were recorded for virtual viewing. Employee Assistance Program (EAP) utilization increase 2-3X among employee groups that attended the sessions. This aligned with our observation that many staff who needed help were previously reticent from shame, fear, language barriers, and other reasons.

During the sessions we answered more than 500 questions on topics including workplace transmission, labor practices that shed light on underlying power dynamics, and societal factors about COVID-19 rates by race and zip code. Sessions were designed to mitigate unhealthy power dynamics and hierarchies. For example, leaders external to these employee groups were brought in as speakers to ensure that everyone felt safe to raise local concerns and that nobody was silenced. A particularly poignant moment came when an environmental services worker commented that she had always wanted to enter the large elegant auditorium where the sessions were held. For some, this was their first time there as an audience member, and not as the person cleaning the room. In 2018, in this same auditorium, 31 all-male portraits of clinical department Chairs—of whom 30 were white and one was Asian—were removed [6]. The homogeneity of these portraits were an undeniable reminder that the U.S. healthcare was and remains a casteocracy. Their removal created essential space for staff to come together across differences to feel heard and respected.

## Conclusions: a small but critical first step

Caste systems formed independently in numerous societies across the world—yet the cross-cultural application of caste as a framework for addressing structural discrimination in U.S. healthcare is a new advance in our understanding. While the COVID-19 pandemic is not behind us, lessons learned from opening communication among employee groups will carry us forward; and our experience provides concrete next steps for organizations—in the U.S. and abroad—to consider in pursuit of equity for their staff and patients. We are expanding health equity and anti-racism efforts at BH, inclusive of voices across job roles, and working with managers to ensure participation. We are listening to the questions raised and genuinely rethinking policies that impact lower-paid employees disproportionately. For example, conversations about a living wage are no longer being whispered in offices and are instead being discussed with conviction and clarity with leaders. Importantly, our work is not a solution to the deeper systemic issues of racism, classism, and ultimately casteism. Rather, bridging conversations between those with and without privilege within healthcare organizations is a necessary step that we must take.

## Data Availability

Not applicable.
